# Use of JYNNEOS (Smallpox and Mpox Vaccine, Live, Nonreplicating) for Persons Aged ≥18 Years at Risk for Mpox During an Mpox Outbreak: Recommendations of the Advisory Committee on Immunization Practices — United States, 2023

**DOI:** 10.15585/mmwr.mm7422a3

**Published:** 2025-06-19

**Authors:** Agam K. Rao, Faisal S. Minhaj, Rosalind J. Carter, Jonathan Duffy, Panayampalli S. Satheshkumar, Kevin P. Delaney, Laura A. S. Quilter, Rachel E. Kachur, Catherine McLean, Danielle L. Moulia, David T. Kuhar, Marie A. de Perio, Ian H. Spicknall, Beth P. Bell, Pablo J. Sánchez, Christina L. Hutson, Amanda C. Cohn

**Affiliations:** ^1^Division of High-Consequence Pathogens and Pathology, National Center for Emerging and Zoonotic Infectious Diseases, CDC; ^2^Immunization Services Division, National Center for Immunization and Respiratory Diseases, CDC; ^3^Division of Healthcare Quality Promotion, National Center for Emerging and Zoonotic Infectious Diseases, CDC; ^4^Division of HIV Prevention, National Center for HIV, Viral Hepatitis, STD, and Tuberculosis Prevention, CDC; ^5^Division of STD Prevention, National Center for HIV, Viral Hepatitis, STD, and Tuberculosis Prevention, CDC; ^6^CDC 2022 Multinational Mpox Response; ^7^National Institute for Occupational Safety and Health, CDC; ^8^University of Washington, Seattle, Washington; ^9^Nationwide Children’s Hospital, The Ohio State University College of Medicine, Columbus, Ohio.

**Summary**

**What is already known about this topic?**

CDC provides interim vaccination guidance for self-limited mpox outbreaks; however, a clade IIb outbreak that began in 2022 has had a protracted course, and the risk for U.S. mpox outbreaks has increased.

**What is added by this report?**

In 2023, the Advisory Committee on Immunization Practices (ACIP) recommended JYNNEOS (smallpox and mpox vaccine, live, nonreplicating) for persons aged ≥18 years who are at risk for mpox during any mpox outbreak and who are at risk for mpox during the ongoing clade IIb outbreak.

**What are the implications for public health practice?**

ACIP recommends JYNNEOS during outbreaks to improve vaccination coverage and limit the scope of outbreaks. As of 2025, the clade IIb outbreak has continued; the need for vaccinating persons at risk will be reassessed as the outbreak evolves.

## Abstract

Since the worldwide eradication of smallpox in 1980, orthopoxvirus vaccines had been used nearly exclusively by persons at risk for occupational exposure to orthopoxviruses, including *Monkeypox virus*, the virus that causes mpox. However, during recent years, the epidemiology of mpox has been changing in countries where the animal reservoirs are believed to live and where endemic transmission has been known to occur for decades. CDC issues outbreak-specific vaccination recommendations based on the epidemiology at the time specific cases or clusters are identified; however, because of the increased risk for U.S. mpox outbreaks, the Advisory Committee on Immunization Practices (ACIP) reviewed results from a previously performed modified Grading of Recommendations Assessment, Development, and Evaluation of the 2-dose JYNNEOS (smallpox and mpox vaccine, live, nonreplicating) vaccination series and an Evidence to Recommendations (EtR) framework addressing multiple domains (e.g., benefits, harms, and target population values and preferences). Based on this assessment, ACIP recommended the use of JYNNEOS (a live, replication-deficient vaccinia virus vaccine) for persons aged ≥18 years at risk for mpox during an mpox outbreak (irrespective of clade). Because the cause of future mpox outbreaks and the populations affected by these outbreaks remain uncertain, public health authorities will continue to issue outbreak-specific vaccination guidance when outbreaks occur. A clade IIb mpox outbreak that began in 2022 continued to cause substantial morbidity and mortality >1 year later. Although CDC had issued outbreak-specific vaccination guidance, it was anticipated that the outbreak would be protracted. For this reason, ACIP reviewed a second EtR framework about outbreaks and in 2023 recommended JYNNEOS for persons aged ≥18 years at risk for acquiring mpox during the multinational clade IIb outbreak. As of 2025, cases continue to occur; however, the future need for the recommendation will be reassessed as the outbreak evolves. Mpox vaccination is not routinely recommended for health care personnel during mpox outbreaks, including during the ongoing clade IIb outbreak.

## Introduction

### *Monkeypox virus* and Mpox Disease

Mpox is a zoonotic infection caused by *Monkeypox virus* (MPXV), a double-stranded DNA virus in the *Orthopoxvirus* genus. The disease is endemic in certain West and Central African countries, particularly in remote and forested areas, where the (as yet undetermined) animal reservoirs are believed to live. Infection is spread from person-to-person via direct contact with infectious lesions (including during sex), respiratory secretions, and fomites; infection can result in deep-seated, well-circumscribed, and often painful lesions that can involve various parts of the body including palms and soles. In endemic countries, mpox can spread from infected animals to humans. The first human mpox case was identified in the Democratic Republic of the Congo in 1970 (*1*) and was initially confused with smallpox, a disease also caused by an orthopoxvirus (*Variola virus*), but that was globally eradicated by 1980 (*2*). Two clades (subtypes) of mpox are recognized: clade I (endemic in the Central African Republic, the Democratic Republic of the Congo, Gabon, the Republic of the Congo, and part of Cameroon), and clade II (endemic in Côte d’Ivoire, Liberia, Nigeria, Sierra Leone, and part of Cameroon). Each clade has been further categorized into subclades because of mutations or deletions in the genome (*3*,*4*).

### U.S. Mpox Cases Before and During 2022

During 2003, the first mpox outbreak outside of Africa occurred in the United States. The outbreak was caused by clade IIa^†^ MPXV and resulted in 47 human cases in six midwestern states, all of which were associated with pet prairie dogs that had previously been housed with small mammals imported from West Africa.^§^ Outbreaks associated with exposure to MPXV-infected animals have not reoccurred in the United States; however, other types of mpox outbreaks have occurred. Twice during 2021, unrelated clade IIb cases were recognized among travelers from a country with endemic MPXV (*5*,*6*). No secondary cases occurred, but close contacts were monitored for 21 days and postexposure vaccinations considered. In May 2022, a global outbreak caused by clade IIb MPXV began, disproportionately affecting certain gay, bisexual, and other men who have sex with men (MSM) (*7*). Vaccinations were recommended for pre- and postexposure prophylaxis but unlike other mpox outbreaks in the United States, this outbreak has had a protracted course; to date, there have been approximately 35,000 U.S. cases,^¶^ and long-term sequelae and deaths have been reported (*8*,*9*). 

Coincident with U.S. outbreaks, mpox epidemiology has been changing in countries where the virus is endemic. Cases are 1) no longer restricted to remote and isolated regions, 2) occurring in higher numbers than in previous years, and 3) occurring in certain countries that have not reported a single human MPXV infection in decades (*10*). Reasons for the changes have been hypothesized to include deforestation, demographic changes, population movement, and waning protection after cessation of routine smallpox vaccination (*1*). These epidemiologic patterns have increased the risk for future mpox outbreaks, including in the United States.

### Currently Licensed Orthopoxvirus Vaccines and Previous Vaccination Recommendations

Two orthopoxvirus vaccines are currently licensed in the United States. ACAM2000** is a live, replication-competent vaccinia virus vaccine licensed in 2007; JYNNEOS (smallpox and mpox vaccine, live, nonreplicating),^††^ is a live, replication-deficient vaccinia virus vaccine licensed in 2019. In 2022, ACIP had recommended JYNNEOS, as an alternative to ACAM2000, for persons aged ≥18 years at risk for occupational exposure to orthopoxviruses (*11*). The recommendations were not limited to MPXV and were developed before the 2022 clade IIb outbreak, when U.S. cases were sporadic and fewer persons were at risk for MPXV exposure. A modified Grading of Recommendations Assessment, Development, and Evaluation (GRADE) that had been performed at that time compared JYNNEOS (the more recently licensed vaccine) to ACAM2000 (a derivative of the vaccine used to eradicate smallpox). There were no ACIP recommendations about mpox outbreaks; however, CDC issued interim vaccination recommendations at the time a specific case or cluster of cases was identified. A single U.S. case was considered an outbreak because of the rarity of these occurrences and the substantial resources needed to investigate and offer vaccinations (*5*); however, outbreaks were typically short in duration, so vaccination recommendations were also typically short term. JYNNEOS was available for the first time in the United States during the 2022 outbreak and has a more favorable safety profile and fewer contraindications than does ACAM2000 (*11*); for this reason, JYNNEOS has been the vaccine used nearly exclusively during the 2022 outbreak.

### Consideration for JYNNEOS Use During Mpox Outbreaks

Because of increased risk for mpox outbreaks in the United States, ACIP began considering data about the use of JYNNEOS for persons aged ≥18 years at risk for mpox during future mpox outbreaks. The populations at risk differ depending on the epidemiology of a specific outbreak; therefore, public health authorities will continue to issue outbreak-specific guidance, including the populations for whom vaccinations are recommended. However, unlike other U.S. mpox outbreaks, the specific clade IIb outbreak that began in 2022 had continued to cause substantial morbidity and mortality more than 1 year after CDC had recommended JYNNEOS; in addition, only one in four persons recommended to receive the vaccine had received both JYNNEOS doses.^§§^ Anticipating a more protracted outbreak than has occurred during previous U.S. outbreaks, ACIP also considered an outbreak-specific recommendation about use of JYNNEOS for persons aged ≥18 years at risk during that specific outbreak.

## Methods

### ACIP Mpox Work Group

The ACIP Mpox Work Group was constituted to review available evidence (e.g., vaccine effectiveness, safety, and mpox epidemiology); it comprised experts in diverse disciplines, including laboratory, public health, regulatory affairs, preparedness, and various clinical topics (e.g., immunology, vaccine safety, vaccination strategy, infection control, worker safety, occupational health, HIV and other sexually transmitted infections, mpox, obstetrics and gynecology, and pediatrics). Federal partners represented multiple U.S. agencies. During September 30, 2022−October 25, 2023, the work group held 30 weekly or biweekly teleconferences to review the scientific evidence.

### Recommendation Considerations

The work group reviewed the 2022 GRADE assessment findings and considered domains within the Evidence to Recommendations (EtR) framework (a process for transparently describing information considered in moving recommendations from evidence to decisions).^¶¶^ Data were considered for use of JYNNEOS for persons aged ≥18 years 1) at risk for acquiring mpox during any mpox outbreak and 2) at risk during the ongoing global clade IIb outbreak. Evaluated domains included benefits and harms, target population values and preferences, and issues of resource use, acceptability to stakeholders, feasibility of implementation, and anticipated impact on vaccine access. In preparation for a vote, ACIP considered these data and newly collected information from the 2022 outbreak. ACIP also reviewed language about the use of JYNNEOS for persons at occupational risk for exposure to MPXV during an mpox outbreak and considerations for future mpox outbreaks.

## Summary of Findings and Rationale for Recommendations

No clinical disease endpoints are available comparing the effectiveness of vaccines against mpox, but prelicensure data involved geometric mean titers and seroconversion data. The 2022 GRADE review*** evaluated these data from three randomized controlled studies and 15 observational studies. After considering the published studies in GRADE, the work group estimated with moderate certainty that the 2-dose JYNNEOS primary series provides a small increase in disease prevention against MPXV compared with that provided by ACAM2000.^†††^ The work group also had low certainty that fewer serious adverse events occur after the JYNNEOS primary series compared with those after the ACAM2000 primary series and that fewer events of myopericarditis occur after the JYNNEOS primary series than after the ACAM2000 primary vaccination. Based on the sum total of their assessment, including the EtR frameworks,^§§§^^,^^¶¶¶^ ACIP voted unanimously in favor of two recommendations.

## Recommendations

On February 22, 2023, ACIP voted to recommend the 2-dose JYNNEOS vaccination series**** for persons aged ≥18 years who are considered to be at risk for mpox during an mpox outbreak.^††††^ On October 25, 2023, ACIP voted to recommend the 2-dose JYNNEOS vaccination series for persons aged ≥18 years who are at risk for acquiring mpox during the ongoing clade IIb outbreak that began in 2022. For the latter vote, persons at risk included 1) MSM^§§§§^ who, during the past 6 months, have had or anticipate experiencing at least one of the following: a new diagnosis of one or more sexually transmitted infections, more than one sex partner, sex at a commercial sex venue, or sex in association with a large public event in a geographic area where mpox transmission is occurring; 2) sexual partners of persons who have any of these risk factors; and 3) persons who anticipate experiencing any of these risk factors.^¶¶¶¶^

## Clinical Considerations

Clinical considerations have been communicated on the CDC website since the start of the multinational clade IIb outbreak in 2022. These considerations were also reviewed by ACIP and included in this report.

### Vaccine Effectiveness

Like other licensed orthopoxvirus vaccines, JYNNEOS contains vaccinia virus, a less virulent orthopoxvirus than either MPXV or variola virus (the causative agent of smallpox). Owing to a high level of protein identity among orthopoxviruses, vaccinia virus vaccines elicit antibodies that provide cross-protection against other orthopoxviruses, including MPXV; this cross-protection was the foundation for the successful global smallpox eradication campaign (*2*). Vaccinia virus and MPXV have a high level (>90%) of nucleotide identity (*12*), and real-world data from the clade IIb outbreak demonstrate vaccine effectiveness (VE) of the 2-dose series ranging from 66% to 89% (*13*–*16*). VE is unlikely to differ across mpox clades because JYNNEOS is a whole-virus vaccine, which elicits an immune response to many vaccinia viral proteins (not to just a subset of viral proteins, as might occur with subunit vaccines). VE might depend on the route of exposure (e.g., mucosal versus other), frequency of exposure, and level of immunocompromise of affected persons. Infections despite vaccination could occur; however, JYNNEOS prevented or decreased the severity of many infections during the ongoing clade IIb outbreak and is expected to be similarly effective during future outbreaks (irrespective of clade).

### Population Considerations

Future mpox outbreaks might differ epidemiologically by populations affected, numbers of cases, and types of activities for which vaccination is indicated. Because of this inherent variability, public health authorities will issue guidance specific to each outbreak. Vaccination might be advised for preexposure or postexposure protection, for a few persons or many persons, and for persons with only certain exposures or risk factors (e.g., medical, behavioral, or occupational). The specific vaccination recommendations will depend on the epidemiology of the outbreak. For the ongoing clade IIb outbreak, the epidemiology is well understood, and for this reason, ACIP was able to specify persons at risk. However, as epidemiology for this outbreak evolves, public health authorities will continue to issue additional guidance. As of 2025, cases, including deaths, continue to occur. To avoid potential stigma associated with affirming risk factors during the ongoing outbreak, clinicians should consider vaccinating persons who request vaccination (i.e., self-attest to vaccine eligibility) without requiring specification of eligibility criteria. Clinicians and public health authorities should be aware that sexual partners of MSM with a new diagnosis of one or more sexually transmitted infections, more than one sex partner, sex at a commercial sex venue, or sex in association with a large public event in a geographic area where mpox transmission is occurring are recommended to be vaccinated. Such persons might include women. However, MSM without risk factors (e.g., those in a monogamous relationship) are not among the population recommended to be vaccinated.

JYNNEOS is contraindicated in persons with a history of a severe allergic reaction (e.g., anaphylaxis) after a previous JYNNEOS dose or to any component of the vaccine (*17*). Similar to other vaccines, JYNNEOS might be less effective in severely immunocompromised persons, but it has been shown to be safe and immunogenic in persons with well-controlled HIV, atopic dermatitis, eczema, or other exfoliative skin conditions (*18*,*19*). No human data regarding safety of JYNNEOS administration during pregnancy or breastfeeding are available; however, JYNNEOS is a nonreplicating vaccine, and data from animal models, including rats and rabbits, have shown no evidence of harm to a developing fetus (Table 1). CDC does not recommend vaccination for any persons who have recovered from mpox or any other orthopoxvirus infection because recovery from MPXV infection (regardless of clade) likely confers protection from either clade of mpox. Persons who have recovered from mpox can experience reinfection; however, CDC surveillance data suggest this is very rare. Surveillance data through June 2025 suggest that reinfections have occurred in <0.001% of U.S. persons who previously had mpox. In these rare instances, the second infection was generally milder than the initial infection.*****

**TABLE 1:** Clinical considerations for use of JYNNEOS* in special populations during an mpox outbreak — United States, 2025

Population	Clinical considerations
Persons with atopic dermatitis, eczema, or other exfoliative skin conditions	• No precautions needed.
	• Studies described in the package insert have indicated JYNNEOS is safe and effective in these circumstances.
Persons with immunocompromising conditions^†^	• No precautions needed.
	• JYNNEOS is safe in these persons because although it is a live virus vaccine, the virus is nonreplicating; it therefore acts like a nonlive vaccine but similar to other vaccines, JYNNEOS might be less effective in persons with severe immunocompromise.
	• Affected persons should be counseled so that preventing exposures remains a high priority regardless of vaccination status.
Pregnant women	• Available data on JYNNEOS administered during pregnancy are insufficient to determine vaccine-associated risk in pregnancy; however, the package insert describes data involving animal models (e.g., rat and rabbit models) that have shown no evidence of harm to the developing fetus.
Breastfeeding women	• The safety and efficacy of JYNNEOS during breastfeeding have not been evaluated.
	• No studies have evaluated whether JYNNEOS affects milk production or safety to breastfed infants. However, because JYNNEOS is replication-deficient, it likely does not present a risk of transmission to breastfed infants and can be administered to the mother if vaccination is indicated based on risks.
Persons aged <18 years^§^	• Data currently do not indicate any safety signals.
	• Vaccination is permitted for children aged <18 years who are at risk for mpox.
	• VIGIV (purified immunoglobulin from persons vaccinated with ACAM2000) should be considered in lieu of JYNNEOS if postexposure vaccination is indicated for infants aged <6 months.
	• ACIP is continuing to assess available data and will make changes to recommendations as needed.

**Abbreviations:** ACIP = Advisory Committee on Immunization Practices; VIGIV = vaccinia immune globulin intravenous.

* Package Insert - JYNNEOS (Refrigerator) | FDA

^†^ Altered Immunocompetence | Vaccines & Immunizations | CDC

^§^ CDC recommendations for use of JYNNEOS during mpox outbreaks for persons aged <18 years is outlined at Interim Clinical Considerations for Use of Vaccine for Mpox Prevention in the United States | Mpox | CDC.

### Health Care Personnel and Laboratorians

For decades, ACIP has recommended that some U.S. persons at occupational risk for exposure to orthopoxviruses receive preexposure vaccination (*20*). Most of these persons have been research laboratory personnel who work with orthopoxviruses; however, some clinical laboratory personnel who work in Laboratory Response Network laboratories (a network of domestic and international laboratories established to respond to biologic and chemical threats and emerging infectious diseases^†††††^) were included for smallpox preparedness, and since the early 2000s, when the concern for biothreats (e.g., due to anthrax) was at its peak, some jurisdictions began maintaining limited cadres of vaccinated health care personnel as well.^§§§§§^

At the time the 2022 ACIP recommendations for JYNNEOS were being developed, mpox cases rarely occurred in the United States, and data regarding transmission in health care settings were primarily from countries with endemic MPXV, where personal protective equipment (PPE) is inconsistently available. A single occupationally acquired case had been reported in a health care provider in the United Kingdom; however, this case was associated with inadequate PPE (*21*). 

With the onset of the 2022 outbreak, transmission of mpox in U.S. health care settings was evaluated. Few occupationally acquired cases occurred among health care personnel (fewer than 25 cases, accounting for <0.08% of all U.S. cases), and no cases among laboratorians. Because infection prevention and control practices were found to be effective in preventing transmission, CDC has not routinely recommended vaccination of clinical laboratory personnel or health care personnel who care for patients during the ongoing outbreak. ACIP agreed that the 2022 recommendations regarding use of JYNNEOS for persons at occupational risk for orthopoxvirus infections apply for persons at risk whether or not an active mpox outbreak is occurring. However, for health care personnel and clinical laboratorians at occupational risk exclusively during an mpox outbreak, the committee concurred that data support JYNNEOS not being routinely recommended. Vaccination of a small number of these persons could be considered on a case-by-case basis if site- and activity-specific biosafety risk assessments during an outbreak suggest that vaccination is warranted; however, these are expected to be rare (Table 2).

**TABLE 2:** Advisory Committee on Immunization Practices preexposure vaccine recommendations for persons at occupational risk for exposure to orthopoxviruses only during an mpox outbreak, including the clade IIb outbreak that began in 2022 — United States, 2025

Population	Recommendation on a case-by-case basis
Health care personnel who care for patients infected with mpox	• Recommended infection prevention and control practices are effective in preventing transmission.
	• ACIP recommends use of JYNNEOS (as an alternative to ACAM2000) based on shared clinical decision-making, i.e., vaccination can be offered based on site- and activity-specific biosafety risk assessments (e.g., inadequate availability of personal protective equipment during humanitarian missions for mpox in endemic countries).
Clinical laboratory personnel who handle specimens^*^ that during an mpox outbreak, might have a higher possibility of containing replication-competent *Monkeypox virus*	• ACIP recommends use of JYNNEOS (as an alternative to ACAM2000) based on shared clinical decision-making.
	• Recommended infection prevention and control practices are effective in preventing transmission.

**Abbreviation**: ACIP = Advisory Committee on Immunization Practices.

* Specimens include lesion material, throat swabs, oral swabs, and rectal swabs. Science Brief: Detection and Transmission of Mpox (Formerly Monkeypox) Virus During the 2022 Clade IIb Outbreak | CDC Archive

### Vaccination Schedule and Duration of Protection

JYNNEOS is recommended as a 2-dose subcutaneous vaccination series, with the second dose administered 28 days after the first. Similar to other multidose vaccines, the second dose could be administered up to 4 days^¶¶¶¶¶^ before the recommended 28-day interval (i.e., 24–27 days after the first dose). If the second dose is not administered during the recommended interval, it should be administered as soon as possible; however, there is no need to restart the series if the interval between doses is prolonged (e.g., >1 year). The duration of protection after the 2-dose series is still being studied, but recently published data indicate protection might be >5 years (*22*). At this time, persons who have been vaccinated with the 2-dose JYNNEOS series do not require an additional dose, nor do they need to be revaccinated during a future outbreak. Although the 2-dose JYNNEOS series may not be as effective in severely immunocompromised persons, it is not known whether additional doses will improve effectiveness; in addition, some data have suggested that more than 2 doses may cause increased reactogenicity (*22*) and for this reason, additional doses are not recommended. As more data become available, CDC might provide additional guidance.

### Timing of Administration of Other Vaccines and of Immunoglobulin Products

JYNNEOS is a live virus vaccine. However, because the vaccinia virus component is nonreplicating, it is managed in nearly every situation as if it were a nonlive vaccine. Unlike other live virus vaccines, no minimum interval is required between receipt of JYNNEOS and other vaccines; however, at this time, theoretical considerations regarding temporal proximity of administration of JYNNEOS and COVID-19 vaccines, and JYNNEOS and vaccinia immune globulin intravenous (VIGIV), are recognized. Although JYNNEOS has not been reported to be associated with myopericarditis, ACAM2000 (a live, replication-competent smallpox and mpox vaccine) is known to be associated with myocarditis.****** Because some COVID-19 vaccines have also been associated with myocarditis,^††††††^ persons (particularly adolescent and young adult males) who are recommended to receive COVID-19 and JYNNEOS vaccines might consider waiting 4 weeks between vaccines out of an abundance of caution. If there is a need for VIGIV to be administered in close temporal proximity to JYNNEOS vaccination, CDC should be consulted for case-specific guidance^§§§§§§^ (Table 3).

**TABLE 3:** Clinical considerations for temporal administration of other vaccines and of immunoglobulin products in relation to JYNNEOS vaccine administration*

Vaccine or immunoglobulin	Guidance
**Vaccine**	
Live, replicating virus vaccines (e.g., yellow fever, measles, and varicella virus vaccines)	• No required interval between JYNNEOS vaccine and live, replicating virus vaccines, because unlike other live virus vaccines, JYNNEOS does not replicate to induce an immune response.
	• For the purposes of planning administration of other vaccines, JYNNEOS may be considered similar to nonlive virus vaccines.
COVID-19 vaccines	• No required minimum interval between receiving any COVID-19 vaccine and JYNNEOS vaccine (e.g., for mpox prevention), regardless of which vaccine is administered first.
	• Persons (particularly adolescent and young adult males) who are recommended to receive both vaccines might consider waiting 4 weeks between vaccines, because of the observed risk for myocarditis and pericarditis after receipt of ACAM2000 orthopoxvirus vaccine and COVID-19 vaccines and the hypothetical risk for myocarditis and pericarditis after JYNNEOS vaccine.
	• If a patient’s risk for mpox or severe disease due to COVID-19 is increased, administration of JYNNEOS and COVID-19 vaccines should not be delayed.
	• This guidance might be revised if the concern for myocarditis and pericarditis abates.
**Immunoglobulin products**	
Antibody containing preparations (e.g., blood products, IVIG) except VIGIV	• No minimum interval between most immune globulins and JYNNEOS vaccine; the former are not associated with mpox prevention but might be administered because of other medical problems.
	• Antibodies to measles and varicella are high in immune globulin products; administration of these in close temporal proximity to the measles and varicella live virus vaccines can prevent the vaccine virus from entering cells and being effective; however, unlike for measles and varicella, antibodies to orthopoxviruses including *Monkeypox virus,* are believed to be low in most antibody containing products, including during the ongoing outbreak.
VIGIV (purified immunoglobulin from persons vaccinated with ACAM2000)^†,§^	• VIGIV is the only known antibody-containing preparation that could potentially interfere with JYNNEOS vaccine. This is because antibody in VIGIV might interfere with entry of the vaccine virus into cells, As a live virus vaccine, entry into cells is essential to effectiveness.
	• Because VIGIV could interfere with immune response to JYNNEOS necessitating an additional JYNNEOS dose at a later time, VIGIV should not be administered in temporal proximity to JYNNEOS, and JYNNEOS should be delayed if VIGIV was recently administered. The duration for which it should be delayed is currently unknown. CDC can be consulted for case-specific guidance.
	• During outbreaks, it is acceptable for VIGIV and JYNNEOS to have been administered in temporal proximity (e.g., if JYNNEOS vaccine was administered to a patient as postexposure prophylaxis but the patient went on to develop a severe manifestation of mpox for which VIGIV is recommended).
	• Public health authorities oversee access to VIGIV and can provide additional guidance if indicated.

**Abbreviations**: IVIG = intravenous immune globulin; VIGIV = vaccinia immune globulin intravenous.

* JYNNEOS is a live virus vaccine but because it is replication-deficient, guidance differs from that for other live virus vaccines (e.g., yellow fever, measles, and varicella vaccines)

^†^ VIGIV is maintained by the U.S. Department of Health and Human Services’ Center for the Strategic National Stockpile and only available under certain circumstances and via consultation with CDC’s on-call poxvirus subject matter experts (CDC Emergency Operations Center: 404-639-3311). Indications for VIGIV are outlined in the Investigational New Drug protocol. Expanded Access IND Protocol: Use of Vaccinia Immune Globulin Intravenous (VIGIV, CNJ-016) for Treatment of Human Orthopoxvirus Infection in Adults and Children | CDC

^§^ Interim Clinical Treatment Considerations for Severe Manifestations of Mpox — United States, February 2023 | MMWR | CDC

### Strategies for Consideration During Outbreaks

During the 2022 U.S. outbreak of clade IIb MPXV, initial demand for vaccination was high, and supplies were limited. To address this shortage, intradermal administration of JYNNEOS was advised as a dose-sparing strategy; intradermal administration required one fifth of the subcutaneous dose and assessments indicated VE comparable to JYNNEOS administered subcutaneously (*14*). Although the intradermal vaccination technique is similar to that used for application of tuberculin skin tests, not all providers were comfortable with this technique. In addition, intradermal JYNNEOS vaccination was associated with a visible nodule or hyperpigmentation at the site of administration, which was stigmatizing for some persons. 

Individual jurisdictions implemented measures including mass vaccination sites and other efforts to make vaccines available to communities with either a high mpox incidence or limited access to health care. Some jurisdictions prioritized first doses and delayed administration of the second dose until adequate supplies were available (*23*). 

At this time, there is an abundance of JYNNEOS vaccine supply; therefore, vaccine doses should be administered subcutaneously. However, if a shortage occurs, JYNNEOS can be administered intradermally. Regardless of vaccine supply and strategy, decisions about vaccine administration during an outbreak should ensure fair and prudent distribution of doses. Vaccinated persons should be advised that peak antibody response is achieved 2 weeks after receipt of the second dose, but that even a single dose provides some protection (*13*–*16*). Vaccinations should be provided along with counseling that breakthrough infections could still occur and the importance of other prevention strategies.

### Reporting Adverse Events

Adverse events following vaccination can be reported to the Vaccine Adverse Event Reporting System (VAERS). Reporting is encouraged for any clinically significant adverse event, even if it is unclear whether the vaccine caused the event. Information on how to submit a report to VAERS is available at Vaccine Adverse Event Reporting System (VAERS) or by telephone at 1-800-822-7967.

### Future Research

Because the proportion of immunocompromised persons has increased in the United States (*24*), information about VE of JYNNEOS among severely immunocompromised persons (e.g., persons with advanced HIV) will be critical to guiding future recommendations. In addition, if more mpox outbreaks occur in the United States, it will be important to know whether there is durable protection after JYNNEOS vaccination or after resolved infection, and if not, when a booster dose might be needed. Because JYNNEOS behaves like a nonlive virus vaccine and is recommended as a 2-dose series, its role as postexposure prophylaxis is poorly understood; studies are ongoing to understand VE of JYNNEOS postexposure vaccination.

## Footnotes

* These authors contributed equally to this report.

† Subclade designations were made after the global clade IIb outbreak in 2022. When the newer sequences were identified as clade IIb, the previous ones were retrospectively designated clade IIa.

§
Past U.S. Cases and Outbreaks | Mpox | CDC

¶
U.S. Case Trends: Clade II Mpox | Mpox | CDC

**
Package Insert - ACAM2000 | FDA

††
Package Insert - JYNNEOS (Refrigerator) | FDA

§§
JYNNEOS Vaccine Coverage by Jurisdiction | Mpox | Poxvirus | CDC

¶¶
Evidence-Based Recommendations for ACIP | ACIP | CDC

***
Grading of Recommendations, Assessment, Development, and Evaluation (GRADE): Use of JYNNEOS (orthopoxvirus) vaccine primary series for research, clinical laboratory, response team, and healthcare personnel (Policy Questions 1 and 2) | ACIP | CDC

††† Although this reflects the findings of the analysis, basic science data which are not included in GRADE supports that ACAM2000 would likely be more effective in prevention of mpox (or smallpox) than JYNNEOS.

§§§
Evidence to Recommendations 1

¶¶¶
Evidence to Recommendations 2

**** Dose 2 should be administered 28 days after dose 1.

†††† Public health authorities will determine whether an mpox outbreak is occurring; a single case might be considered an mpox outbreak at the discretion of public health authorities. Other circumstances in which a public health response might be indicated include ongoing risk for introduction of mpox into a community because of disease activity in another geographic area.

§§§§ Wording previously published (Recommended Adult Immunization Schedule for ages 19 years or older-2024 U.S. | CDC) has been amended to comply with Executive Order 14168. Defending Women from Gender Ideology Extremism and Restoring Biological Truth to the Federal Government – The White House

¶¶¶¶ Because there might be stigma associated with affirming risk factors, clinicians should consider vaccinating persons who request vaccination (i.e., self-attest to vaccine eligibility) without requiring specification of the criterion that deems eligibility.

*****
Interim Clinical Considerations for Use of Vaccine for Mpox Prevention in the United States | Mpox | CDC

†††††
About the Laboratory Response Network | The Laboratory Response Network Partners in Preparedness | CDC

§§§§§
CDC interim guidance for revaccination of eligible persons who participated in the US civilian smallpox preparedness and response program

¶¶¶¶¶
Timing and Spacing of Immunobiologics | Vaccines & Immunizations | CDC

******
ACAM2000 (Smallpox Vaccine) Questions and Answers | FDA

††††††
CDC. Clinical considerations: myocarditis after COVID-19 vaccines

§§§§§§ VIGIV is maintained by the U.S. Department of Health and Human Services’ Center for the Strategic National Stockpile and only available under certain circumstances and via consultation with CDC’s on-call poxvirus subject matter experts (CDC Emergency Operations Center: 404-639-3311). Investigational New Drug protocol | CDC
